# Supplementary material for: Effects of antennal segments defects on blood-sucking behavior in Aedes albopictus
Source: PLoS One. 2023 Aug 10;18(8):e0276036. doi: 10.1371/journal.pone.0276036 (PMC10414602; doi:10.1371/journal.pone.0276036)
Supplement: Attachment — Submitted filename: MOSSY-6-s13071-022-05259-x.pdf [file pone.0276036.s002.pdf]

Parasites & Vectors

RESEARCH Open Access

Genome-wide identification and expression profiling of odorant receptor genes in the malaria vector *Anopheles sinensis*

Zhengbo He*, Zhengrong Yu, Xingfei He, Youjin Hao, Liang Qiao, Shihui Luo, Jingjing Zhang and Bin Chen*

Abstract

Background: The olfactory system plays a crucial role in regulating insect behaviors. The detection of odorants is mainly mediated by various odorant receptors (ORs) that are expressed in the dendrites of olfactory neurons of chemosensilla. *Anopheles sinensis* is a major malaria vector in Eastern Asia and its genome has recently been successfully sequenced and annotated. In this study, we present genome-wide identification and expression profiling of OR genes in different chemosensory tissues of *An. sinensis*.

Methods: The OR genes were identified using the available genome sequences of *An. sinensis*. A series of bioinformatics analyses were conducted to investigate the structure, genome distribution, selective pressure and phylogenetic relationships of OR genes, the conserved domains and specific functional sites in the OR amino acid sequences. The expression levels of OR genes were analyzed from transcriptomic data from *An. sinensis* antennae, proboscis and maxillary palps of both sexes.

Results: A total of 59 putative OR genes have been identified and characterized in *An. sinensis*. This number is significantly less than that in *An. gambiae*. Whether this difference is caused by the contraction or expansion of OR genes after divergence of the two species remains unknown. The RNA-seq analysis showed that *AsOR*s have obvious tissue- and sex-specific expression patterns. Most *AsORs* are highly expressed in the antennae and the expression pattern and number of *AsOR*s expressed in antennae are similar in males and females. However, the relative levels of *AsOR* transcripts are much higher in female antennae than in male antennae, which indicates that the odor sensitivity is likely to be increased in female mosquitoes. Based on the expression patterns and previous studies, we have speculated on the functions of some OR genes but this needs to be validated by further behavioral, molecular and electrophysiological studies. Further studies are necessary to compare the olfactory-driven behaviors and identify receptors that respond strongly to components of human odors that may act in the process of human recognition.

Conclusions: This is the first genome-wide analysis of the entire repertoire of OR genes in *An. sinensis*. Characterized features and profiled expression patterns of ORs suggest their involvement in the odorous reception of this species. Our findings provide a basis for further research on the functions of OR genes and additional genetic and behavioral targets for more sustainable management of *An. sinensis* in the future.

Keywords: *Anopheles sinensis*, Odorant receptor, Genome, Characteristics, Expression pattern

*Correspondence: zhengbohe@cqnu.edu.cn; bin.chen@cqnu.edu.cn
Chongqing Key Laboratory of Vector Insects, Institute of Entomology and Molecular Biology, College of Life Sciences, Chongqing Normal University, Chongqing 401331, People's Republic of China

Background

Anopheles sinensis Wiedemann is an important vector of *Plasmodium vivax* in China and Southeast Asian countries [1, 2]. It can also transmit lymphatic filariasis,

© The Author(s) 2022. **Open Access** This article is licensed under a Creative Commons Attribution 4.0 International License, which permits use, sharing, adaptation, distribution and reproduction in any medium or format, as long as you give appropriate credit to the original author(s) and the source, provide a link to the Creative Commons licence, and indicate if changes were made. The images or other third party material in this article are included in the article's Creative Commons licence, unless indicated otherwise in a credit line to the material. If material is not included in the article's Creative Commons licence and your intended use is not permitted by statutory regulation or exceeds the permitted use, you will need to obtain permission directly from the copyright holder. To view a copy of this licence, visit http://creativecommons.org/licenses/by/4.0/. The Creative Commons Public Domain Dedication waiver (http://creativecommons.org/publicdomain/zero/1.0/) applies to the data made available in this article, unless otherwise stated in a credit line to the data.

Japanese encephalitis virus and *Rickettsia felis* [2]. Malaria outbreaks and re-emergence have only occurred in regions with *An. sinensis* in recent years in China [3, 4]. Therefore, the control of *An. sinensis* is considered to be one of the most effective measures to prevent malaria transmission in China. However, malaria control interventions face many challenges due to the increase in drug resistance in parasites and insecticide resistance in mosquitoes. The impact of these trends on existing control measures should be of great concern and the exploration of new mosquito-centered control strategies should be strengthened.

As in other mosquito species, *An. sinensis* females require a blood meal to complete each gonotrophic cycle, which includes alternate host-seeking, blood-feeding, resting and egg-laying [2]. Vector-host interactions are largely dependent on effective mosquito responses to cues from vertebrate hosts, such as olfaction, vision, mechanical vibration, sound, humidity and thermal cues. Among these cues, olfaction plays a critical role in female behavior (host seeking, blood feeding, oviposition site selection, etc.) and thus directly impacts their vector capacity to transmit pathogens [5]. Therefore, intensive molecular studies on the *An. sinensis* olfactory system will provide the necessary insights required for the development of new strategies to disrupt host-seeking behavior.

Olfactory recognition in insects is a complicated process that is principally accomplished through a series of proteins located in olfactory appendages, such as antennae, proboscis and maxillary palps. These proteins include odorant receptors (ORs), gustatory receptors (GRs), ionotropic receptors (IRs), odorant-binding proteins (OBPs), chemosensory proteins (CSPs), sensory neuron membrane proteins (SNMPs), odorant degrading enzymes (ODEs) and Niemann-Pick protein C2 (NPC2) [6, 7]. The ORs, such as the olfactory receptor co-receptor (Orco) and conventional ligand-binding odorant receptors, play key roles in olfactory behavior [8]. Since the first identification of insect ORs in *Drosophila melanogaster* in 1999, many OR genes have now been identified in species from at least seven insect orders, which include Diptera, Lepidoptera, Hymenoptera, Coleoptera, Homoptera, Orthoptera and Blattodea [9–21]. In mosquitoes, OR repertoires have been identified in *Anopheles gambiae*, *Anopheles darlingi*, *Aedes aegypti*, *Culex quinquefasciatus* and *Aedes albopictus* through genome-wide identification [9, 11, 13, 17, 22]. The number of OR genes vary considerably from 18 in *An. darlingi* [22] to 112 in *Cx. quinquefasciatus* [13] and up to 158 in *Ae. albopictus* [17]. This reflects extensive gene gains and losses over the evolution of mosquito ORs. The OR genes are mainly expressed in antennae and other chemosensory appendages, where they detect various volatile compounds and are involved in diverse olfaction-driven behaviors [11, 23–25].

The ORs do not recognize odor molecules alone but can form heteromeric complexes with Orco. The complex converts chemical signals into electrical signals and transmits nerve impulses to dendrites in olfactory neurons [26]. Studies have shown that ORs are involved in host-seeking, mating, oviposition site searching and other important behaviors in mosquitoes. After mutating the *Orco* gene of *Ae. aegypti*, using ZFN technology, the *Orco* mutant showed a significantly reduced response to honey. In the absence of CO_2, it was unresponsive to human odors and lost its host preference [27]. Further analysis revealed that the host preference of *Ae. Aegypti*, for human odor, was associated with increased expression of *AaegOr4,* which recognizes a human odorant, sulcatone [28]. Reduced levels of *Ae. albopictus AalOrco* result in a significant decrease in host-seeking and confusion in host preference [29]. The *An. gambiae* ORs seem to be narrowly tuned to several odor components that emanate from humans, such as 1-octen-3-ol, 2, 3-butanedione and indole [30]. *Anopheles gambiae AgamOr8* and *Ae. aegypti AaegOr8* are specifically expressed in maxillary palps and respond strongly to 1-octen-3-ol and CO_2 [11, 31, 32]. In addition, studies have shown that a specific OR gene was the target of DEET and other repellents as well as the natural repellent, methyl jasmonate [27, 33–36].

After the release of the genome sequence of *An. sinensis* [37], chemosensory genes, such as CSPs [38], OBPs [39] and IRs [40–42], have been characterized. However, genome-wide identification and analysis of ORs, GRs, ODEs and SNMPs have not been completed. Currently, only 33 OR genes have been identified in *An. sinensis* [37, 43]. However, the number, classification, expression characteristics and functions of OR genes are still unknown. Like other mosquitoes, female *An. sinensis* detect odorants or various chemical cues in their environment, through several receptor genes, to find their hosts and blood supply. Therefore, a comprehensive understanding of the cues that attract mosquitoes to humans and the receptors that detect them will provide the necessary insight into developing new strategies to disrupt host-seeking behavior [27].

In this study, all OR genes were identified using the available genome sequence of *An. sinensis* and a series of bioinformatics analyses were conducted. These included analysis of the structure, genome distribution, selective pressure, phylogenetic relationships, conserved domains and specific functional sites in their amino acid sequences. Expression levels of OR genes were analyzed using transcriptomic data from *An. sinensis* antennae,

proboscis and maxillary palps of both sexes. This study established an information framework for *An. sinensis* OR genes and enriched the data for traditional OR genes, which will facilitate their functional studies and investigations into mechanisms of olfactory-driven behavior in *An. sinensis*.

Methods

Sequence retrieval and identification of OR genes in *An. sinensis*

Two versions of the *An. sinensis* genome were used for the identification of OR genes. One version was downloaded from GenBank (gca_000441895.2). The other was sequenced using the PacBio sequencing approach at the Beijing Genomics Institute (BGI) by Chongqing Normal University (in preparation). The final contigs spanned 245.6 Mb with an N50 contig size of 3.1 Mb. The integrity of gene region was 97%, as evaluated by EST/Unigenes, and the assembly integrity, by BUSCO evaluation, was 97.9%. Due to the high coverage and good quality assembly, this study mainly used the *An. sinensis* genome sequenced by Chongqing Normal University and used the released genome (gca_000441895.2) as a reference. Two sets of transcriptome data were downloaded from the National Center for Biotechnology Information (NCBI) EST database (Accession numbers: SRA073189 and GAFE01000001-GAFE01028133).

To identify orthologous genes that encode ORs in *An. sinensis,* the full-length amino acid sequences of the ORs in *An. gambiae*, *Cx. quinquefasciatus*, *Ae. aegypti* and *D. melanogaster* were sourced from FlyBase, Uniprot and GenBank. These were then used as query sequences to perform a local BLASTp search (*E*-value cutoff of $< 1^{e-5}$) against the *An. sinensis* genome database. In addition, Hidden Markov Model (HMM) searches were conducted against the protein database of *An. sinensis* using the OR protein domain, HMM profile (Pfam02949). The duplicated genes and incomplete sequences were manually removed, and original candidate genes were obtained. The identified AsOR genes were named in accordance with their closest *An. gambiae* homologs to facilitate comparison. Abbreviations (As: *Anopheles sinensis*, Ag: *Anopheles gambiae*, Cx: *Culex quinquefasciatus* and Aa: *Aedes aegypti*) of the species names were used as prefixes to the specific gene name for identification.

Sequence characterization

Molecular weight (MW) and isoelectric point (pI) of ORs were predicted using ExPASy (http://web.expasy.org/protparam/). The transmembrane helices were analyzed using the online server GPCRHMM (http://gpcrhmm.sbc.su.se/) and HMMTOP (http://www.enzim.hu/hmmtop/). Signal peptides and subcellular localization prediction were performed by SignalP4.1 (http://www.cbs.dtu.dk/services/ SignalP/) and WoLF PSORT (https://wolfpsort.hgc.jp/). Conserved domains were analyzed by SMART (http://smart.embl-heidelberg.de/) and CD-search, with the default parameters (http://www.ncbi.nlm.nih.gov/Structure/cdd/ docs/cdd_search. html). The pairwise identity matrix of all ORs was generated by MEGAX and visualized using the pheatmap package in R (https://cran.r-roject.org/web/packages/pheatmap/index.html).

Scaffold location, gene structure and conserved motif analysis

The physical chromosomal location data for each AgOR was downloaded from Supporting Online Material [9] and mapped onto the chromosomes using MapInspect. To map the AsOR genes onto the scaffold, a BLASTN search was conducted against the *An. sinensis* genome. To illustrate the gene structure of AsOR genes, the exon-intron structure, including exon positions and gene length, was constructed using the online Gene Structure Display Server (http://gsds.cbi.pku.edu.cn/). Protein sequence motifs were identified using Multiple En for Motif Elicitation (MEME) (http://meme-suite.org/tools/meme), with the following parameters: number of repetitions: any, the maximum number of motifs: 10, optimum motif width set to > 6 and < 200. The predicted MEME motifs were searched in the Expasy-Prosite database with the ScanProsite server (https://prosite.expasy.org/scanprosite/).

Identification of ortholog pairs in four mosquito species

The OrthoMCL program was applied to identify OR orthologs in four mosquito species. In brief, the BlastP search against Diptera ortholog categories was performed with an e-value of $< 1e^{-10}$. Gene duplication was considered with the following criteria: (1) genes with > 70% coverage of the alignment length; (2) identity > 70% within the aligned region. Tandem duplication was considered when two closely related AsOR genes were located on the same scaffold.

Selective pressure analysis of OR orthologs between *An. sinensis* and *An. gambiae*

The nonsynonymous to synonymous substitution ratios (*Ka*/*Ks*) for OR orthologous pairs in *An. sinensis* and *An. gambiae* were calculated using the yn00 program of PAML 4 [44, 45]. The *Ka/Ks* ratios were used to assess the selection pressure on OR genes and *Ka/Ks* ratio > 1, < 1 or = 1 indicated positive, negative or neutral evolution, respectively.

Phylogenetic analysis of OR genes in four mosquito species

The reconstruction of evolutionary relationships was performed using the amino acid sequence of the conserved OR domains because the flanking regions of the conserved domain were either nonhomologous or too divergent. Pseudogenes and incomplete genes were excluded. Multiple sequence alignments were performed using MAFFT, with the default settings [46]. Positions of ambiguous alignment were removed using the online version of Gblocks, with the "less stringent" options (http://molevol.cmima.csic.es/castresana/Gblocks_server.html). The best substitution model for the alignment was determined using ProtTest serverV3.2.1. Phylogenetic inference for the aligned sequences was conducted using a Maximum-likelihood method, as implemented in RAxMLv8.2.0, with 1000 bootstrap replicates [47]. The phylogenetic tree was viewed and edited using iTOL (http://itol.embl.de/index.shtml).

Insect rearing and sample collection

The *An. sinensis* laboratory population was reared at 28 °C, in 75–80% relative humidity (r.h.) and a light: dark = 12 h:12 h photoperiod at the Institute of Entomology and Molecular Biology, Chongqing Normal University. The larvae were fed on fish food (Tetramin, Melle, Germany) and were maintained at densities of approximately 120 per liter of water. Pupae were collected and allowed to eclose in plastic cages. Adults were reared with a 10% glucose solution and blood-fed on anesthetized mice for approximately 20 min at third after emergence. To obtain mosquitoes for RNA-seq analyses, pupae were sorted by sex, and males and females were kept separately in plastic cages. The emerged mosquitoes were removed from the cage each day to obtain mosquitoes of the same age. Female or male adult mosquitoes at 0, 6, 12 and 18 h on the 3rd day after emergence were collected separately, and the four samples of the same sex were mixed together in equal proportions and placed in petri plates on ice. The antennae, proboscis and maxillary palps were manually dissected under a dissection microscope and stored immediately in RNAlater®-Ice (Ambion, Austin, TX, USA). Approximately 1000 females and males were dissected for each replicate, and three replicates were included for each olfactory tissue. The samples were kept at − 80 °C until total RNA was extracted.

RNA extraction, library preparation and sequencing

The total RNA was isolated from each sample using TRIzol reagent (Invitrogen, Carlsbad, CA, USA), in accordance with the protocol provided by Invitrogen. To remove genomic DNA, the RNA samples were treated with RNase-Free DNase I, following the manufacturer's protocol (Cwbio, Beijing, China). The RNA integrity was assessed using the RNA Nano 6000 Assay Kit of the Bioanalyzer 2100 system, with a minimum integrity number of seven (Agilent, CA, USA). Sequencing libraries were generated using the NEBNext® Ultra™ RNA Library Prep Kit from Illumina® (NEB, USA), following the manufacturer's recommendations. Index codes were added to attribute sequences to each sample. The clustering of the index-coded samples was performed on a cBot Cluster Generation System, using the TruSeq PE Cluster Kit v3-cBot-HS (Illumina), in accordance with the manufacturer's instructions. After cluster generation, the library preparations were sequenced on an Illumina Hiseq platform and 125 bp/150 bp paired-end reads were generated at Novogene Bioinformatics Technology Co., Ltd. (Tianjin, China).

Read mapping and data processing

Clean reads were obtained by removing reads that contained adapters, reads that contained poly-N and low-quality reads from the raw data. The Q20, Q30 and GC content of the clean data were also calculated. After filtering, reads from each sample were mapped to the *An. sinensis* reference genome (AsinS2.6) using Hisat2 v2.0.4 [48], allowing for two base-pair mismatches. We used HTSeq v0.9.1 to count the read numbers mapped to each gene. The fragments per kilobase of transcript per million mapped reads (FPKM) of each gene was calculated based on gene length and the number of mapped reads. Read alignment and expression quantification were performed separately for each sample. The expression levels of the transcripts were expressed as FPKM values of mRNA using Cufflinks v2.2.1 [49] and StringTie v1.3.3 [50]. A value of 1 was added to the FPKM value of each gene, before $\log_2$ transformation, to avoid infinite values. Pearson's correlations were estimated across different tissues, and hierarchical clustering was performed using Multi Experiment Viewer (MeV version 4.9.0).

Identification of chemosensory genes and differential gene expression

To identify candidate chemosensory genes (ORs, IRs, GRs, SNMPs, OBPs and CSPs), the available sequences of OR, IR, GR, SNMP, OBP and CSP proteins from *An. gambiae*, *Ae. aegypti*, *Cx. quinquefasciatus* and *D. melanogaster* were used as queries. The retrieved queries were used to blast against our transcriptomes using tBLASTn, with an e-value cut-off $< 1e^{-5}$. Subsequently, all identified candidate unigenes were manually checked by BLASTx searches against the NCBI Nr database (*e*-value < 1e−5). The ORFs (open reading frames) of candidate chemosensory genes were predicted in the ORF finder tool of the NCBI (https://www.ncbi.nlm.nih.gov/orffinder/). Moreover, each transcript was analyzed by BLAST analysis of

Table 1 Identification and characteristics of *An. sinensis* OR genes

Gene	Scaffold location	ORF length (bp)	AA length	MW (kD)	pI	TM number	Exon number	Transcript
AsOR1	scaffold150:540332:543772	1269	422	49	8.25	6	8	+
AsOR2	scaffold1:506824:508434	1125	374	43	9.17	7	7	+
AsOR3	scaffold46:262985:268589	1173	390	45	8.78	7	8	+
AsOR4	scaffold46:261448:264690	1122	373	43	8.91	6	3	+
AsOR5	scaffold46:260519:262038	1170	389	45	8.73	7	6	+
AsOR6	scaffold15:5845033:5847829	1173	390	44	6.45	6	8	+
AsOrco	scaffold14:22701831:22712003	1437	478	54	8.31	7	8	+
AsOR8	scaffold116:1663019:1664456	1200	399	46	6.75	7	3	+
AsOR9	scaffold49:7034401:7037271	1290	429	50	9.36	7	7	+
AsOR10	scaffold1:529437:530935	1125	374	43	8.36	7	6	+
AsOR11	scaffold55:2028484:2029955	1347	448	50	8.77	7	4	+
AsOR13	scaffold1:2092994:2094445	1161	386	44	7.12	6	5	+
AsOR14	scaffold5:10696239:10697883	1266	421	42	8.47	7	6	+
AsOR16	scaffold1:2096624:2098069	1158	385	44	7.58	5	5	+
AsOR18	scaffold5:10692278:10692850	573	190	–	–	–		ND
AsOR22	scaffold56:2773936:2777792	1164	387	45	7.99	6	5	+
AsOR23	scaffold4:1133159:1134710	1161	386	44	7.5	6	4	+
AsOR24	scafolld9:589896:591445	1197	398	42	8.29	7	5	+
AsOR28	scaffold14:28752212:28754554	1197	398	46	6.6	6	7	+
AsOR29	scaffold1:2102202:2103548	1056	384	45	8.63	7	3	+
AsOR30	scaffold1:2100386:2101733	1161	386	41	7.48	8	4	+
AsOR31	scaffold25:5159352:5160749	1092	363	41	8.74	7	4	+
AsOR32	scaffold25:5344272:5345620	1152	383	43	7.6	6	4	+
AsOR33	scaffold25:1030198:1033992	1203	400	46	7.16	7	6	+
AsOR34	scaffold14:17229952:17231316	1113	370	44	9.14	7	4	+
AsOR35	scaffold25:5196612:5197973	1231	401	45	8.78	7	4	+
AsOR36a	scaffold16:7366107:7367604	1200	399	47	9.28	7	2	+
AsOR36b	scaffold16:7363992:7365407	1200	399	47	9.28	8	2	+
AsOR37	scaffold14:17227440:17228838	1185	394	47	9.47	9	4	+
AsOR38	scaffold25:9882907:9884968	1257	418	49	8.46	7	8	+
AsOR39	scaffold100:3667511:3669156	1260	419	49	8.49	6	6	+
AsOR40	scaffold14:22677686:22679863	1359	456	54	8.41	6	4	+
AsOR41	scaffold16:5533963:5535221	1116	371	43	8.76	7	3	+
AsOR42	scaffold14:8897108:8898493	1173	390	45	6.45	7	4	+
AsOR43	scaffold9:470702:472176	1161	386	44	6.75	8	5	+
AsOR44	scaffold9:473734:475189	1161	386	45	6.06	8	5	+
AsOR45	scaffold14:276476:280805	1158	385	43	8.7	7	1	+
AsOR46	scaffold1:2087486–2089114	1164	387	45	8.66	7	5	ND
AsOR48	scaffold20:3319392:3321000	1182	393	46	8.97	7	4	+
AsOR49	scaffold20:3316974:3318519	1269	422	49	9.35	7	5	+
AsOR51	scaffold5:10696148:10697573	1197	398	46	8.43	7	4	ND
AsOR52	scaffold16:5626401:5627787	1200	399	47	9.5	6	4	+
AsOR54	scaffold5:10598358:10599825	1218	405	47	6.5	7	5	+
AsOR56	scaffold14:34769099:34770620	1203	400	46	8.61	7	5	ND
AsOR57	scaffold14:34771769:34773365	1170	389	45	7.52	7	4	ND
AsOR58	scaffold91:47717:49012	1212	403	46	8.94	7	2	+
AsOR59	scaffold14:16993846:16995214	1218	405	48	8.97	7	3	+
AsOR60	scaffold7:5061115:5062517	1269	422	50	9.12	8	3	+
AsOR61a	scaffold7:4935846:4938118	1242	413	48	6.32	7	4	+

Table 1 (continued)

Gene	Scaffold location	ORF length (bp)	AA length	MW (kD)	pI	TM number	Exon number	Transcript
AsOR61b	scaffold7:4935841:4940099	1269	422	49	8.98	7	3	+
AsOR62	scaffold7:5078106:5079494	1263	420	49	9.27	7	3	+
AsOR63	scaffold7:4954285–4955667	1251	416	50	8.44	7	3	ND
AsOR64	scaffold7:4940417–4941826	981	326	38	8.74	5	3	+
AsOR66	scaffold14:12884846:12886225	1176	391	46	6.45	8	3	+
AsOR68	scaffold2:89904:91961	1170	389	45	8.14	6	4	+
AsOR69	scaffold2:106626:108152	1164	387	45	5.63	7	6	+
AsOR70	scaffold2:109094:110469	1116	371	43	6.16	7	5	+
AsOR76	scaffold14:39976695:39978056	1233	410	47	8.82	8	3	+
AsOR77	scaffold14:39982048:39985415	1146	381	44	8.92	8	2	+

ORF open reading frame, *AA* amino acid, *pI* isoelectric point, *Mw* molecular weight, *ND* not detected, – no data

several databases. Transcripts that showed significant matches with proteins involved in chemosensation were identified. Differential expression analysis was performed using the DESeq R package. The criteria of significant differential expression were $|\log_2 \text{Ratio}| \geq 1$ [51] and False Discovery Rate (FDR) ≤ 0.001[52]. If there was more than one transcript for a gene, the longest transcript was used to calculate its expression level and coverage.

Total RNA isolation and quantitative real time PCR (RT-qPCR)

Total RNA from antennae, proboscis and maxillary palps was extracted using TRIzol Reagent (Invitrogen, Carlsbad, CA, USA). The extracted RNA was quantified and qualified using a NanoDrop ND-1000 spectrophotometer and 2% agarose gel electrophoresis, respectively. The RNA was treated with DNase I (Cwbio, Beijing, China) to remove genomic DNA contamination. Approximately 1 µg of RNA was reverse transcribed into the first-strand cDNA using the SuperScript III RT Kit (Invitrogen, Carlsbad, CA, USA). The RT-qPCR was performed using PrimeScript™ RT Master Mix (Takara, Dalian, China) in a 25-µl system that contained 200 nM forward and reverse primers, 200 µM dNTPs, 2.5U TB Green Premix Ex Taq II and 1 µl cDNA template (approximately 40 ng). The thermal cycling conditions were as follows: 95 °C for 30 s, 40 cycles of 95 °C for 5 s and 60 °C for 30 s. Three biological and three technical replicates were performed for each sample. The data were analyzed by the $2^{-\Delta\Delta CT}$ method [53], and results were expressed as $\log_2$-transformed fold change values. A housekeeping gene, *RpS7*, was used as an internal control. Gene-specific primers that spanned exon-intron boundaries were designed using Primer 5.0 and are listed in Additional file 1: Table S1.

Statistical analysis

All data were represented as means ± SE. The expression data from the RT-qPCR were analyzed using one-way ANOVA for different tissues from males or females, and a Student's *t*-test analysis was performed for the same tissue. Statistical significance was considered at $^*P < 0.05$.

Results and discussion

Identification, nomenclature and characterization of ORs in the *An. sinensis* genome

The analysis of available transcriptomic and genomic data from *An. sinensis* allowed us to identify 59 OR-like sequences. Thirty-three of these *AsORs* have previously been automatically annotated in the *An. sinensis* genome by Zhou et al. [37, 43]. Using Scaffold5, a deduced amino acid sequence fragment that was similar to *An. gambiae AgOR18* was detected near *AsOR51* and showed 46% amino acid identity with the partial sequence of *AgOR18*. However, we were not able to obtain a complete gene, and it is possible that this was a pseudogene. The *AsOR64* gene was provisionally annotated as an incomplete gene because the conserved OR domain was missing. Of the remaining 57 genes, 5 (*AsOR46*, *AsOR51*, *AsOR56*, *AsOR57* and *AsOR63*) did not have transcription support but their amino acid sequences were characteristic of the functional domains, and they shared a high sequence identity (> 30%) with the ORs reported in other mosquitoes and *D. melanogaster* (Additional file 2: Table S2). As a consequence, these five genes were considered to be functional genes. The candidate *An. sinensis* ORs were designated as AsOR1 to AsOR77, based on their orthologous relationship with *An. gambiae* OR genes. Basic information about these genes is provided in Table 1 and Additional file 2: Table S2. The cDNA and protein sequences of the 59 genes are provided in Additional file 3: Table S3.

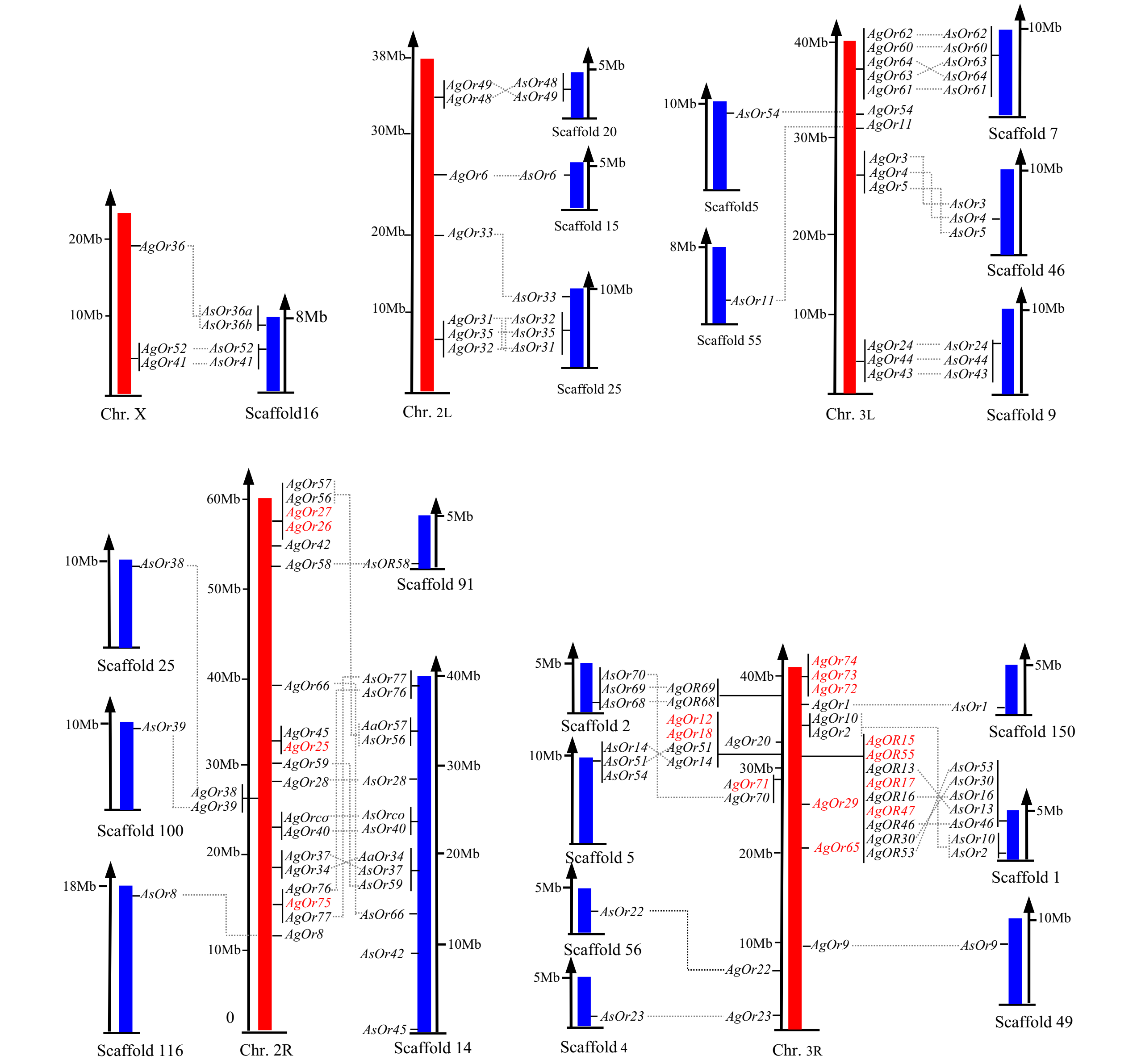

Fig. 1 Comparison of chromosomal or scaffold distribution of *An. sinensis* (in blue) and *An. gambiae* (in red) OR genes. The position of each gene is mapped on the chromosomal or scaffold in Mb scale. Orthologous gene pairs are linked with lines and the unigenes are marked in red

The complete AsORs typically ranged between 351and 429 amino acids (aa), which was similar to ORs of *An. gambiae*, *Cx. quinquefasciatus* and *Ae. Aegypti*. However, AsOrco (477 aa) and AsOR40 (456 aa) were slightly longer (Table 1). All the OR genes were predicted to be located in the plasma membrane. The C-terminus was the most conserved region among AsORs and, in most cases, included a SYS motif near the extreme C-terminus of the conceptual translation. This is a feature that is conserved in *D. melanogaster*, *An. gambiae* and *Ae. aegypti* [9, 11, 54]. The AsOR motifs obtained from the MEME analysis showed that the individual members display a high degree of sequence divergence (Additional file 4: Fig. S1), which was consistent with the requirement for recognition of many odorant molecules [55]. As is the hallmark of all G-protein-coupled receptors (GPCRs) in the chemosensory receptor family, all the AsOR peptides contained multiple transmembrane regions. These regions were relatively conserved, as observed in *An. gambiae*, *Cx. quinquefasciatus* and *Ae. aegypti* (Table 1, Additional file 5: Fig. S2). The membrane orientation predictions showed that most of these mosquito ORs had

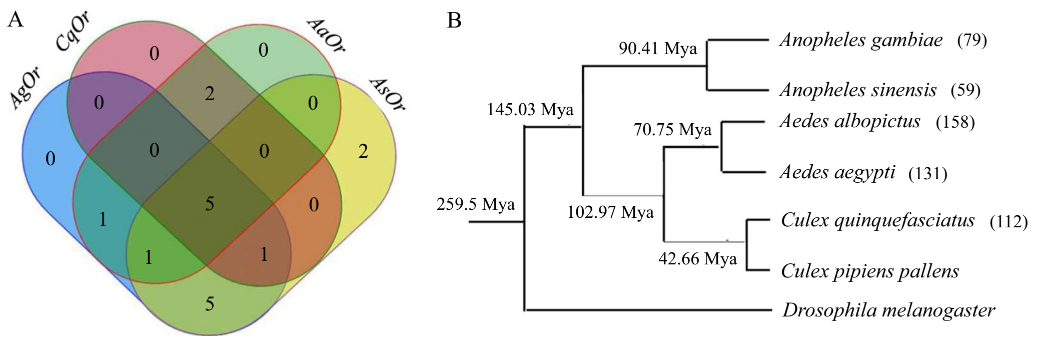

Fig. 2 Venn diagram of orthologous groups of OR genes in four mosquito genomes and the phylogenetic relationship of six important mosquito species. **A** Ortholog categories were analyzed by Blast searches against Diptera orthologs. The numbers displayed in the Venn diagram correspond to the number of ortholog groups. Overlapping regions show the number of ortholog groups shared by two, three or four species. Areas that do not overlap between circles indicate unique genes for each species. **B** The phylogenetic tree was constructed through the taxonomy browser in GenBank. The divergent time was cited from Hao et al. [78]. The numbers in brackets indicate the number of OR genes

an intracellular N-terminus, which was consistent with the known structure of *Drosophila* ORs [8]. In contrast, mouse ORs are predicted to have an extracellular N-terminus [56].

Gene structure, distribution and syntenic analysis of *An. sinensis* ORs

To characterize the structural diversity of *AsOR* genes, their intron-exon organization was analyzed. As shown in Additional file 6: Fig. S3, the majority of *AsORs* contained three to seven exons, while five genes (*AsOR1*, *AsOR6*, *AsOR7* and *AsOR38*) possessed eight exons. The size of most introns ranged from 60 to 100 bp. The largest intron (3789 bp) was found in *AsOrco*. The intron number, intron phase and exon length were highly conserved within the same gene group. Further analysis indicated that these patterns were also highly conserved in *OR* genes of *An. gambiae, Ae. aegypti* and *Cx. quinquefasciatus.*

The genomic organization analysis revealed that 59 OBP genes were distributed across 19 different scaffolds in *An. sinensis*, with an uneven distribution pattern (Fig. 1). Some scaffolds had a high gene density when compared to others. Scaffold 1 possessed the highest density, with four genes covering a region of 500 kb. Scaffold 14 had the largest numbers of AsOR genes (13 genes) but a low density (0.3 gene/M bases). Moreover, multiple AsOR genes were clustered on the same scaffold and were tightly linked as pairs, triplets and larger clusters of up to five genes. Sixteen AsORs existed as single genes (Table 1, Fig. 1). This distribution pattern was similar to the organization of odorant receptors in *An. gambiae* [9] and *Ae. aegypti* [11].

To further explore the evolutionary relationship between AsOR genes and AgOR genes, syntenic mapping was conducted. All *AsORs* were mapped to the corresponding syntenic blocks of the *An. gambiae* genome, which covered five genomic regions of chromosome X, 2R, 2L, 3R and 3L in *An. gambiae* (Fig. 1). Interestingly, 16 *AgOR*s on chromosome 2R and 3R had no orthologous or paralogous genes identifiable in the *An. sinensis* genome, which indicated that gene expansion occurred in the *An. gambiae* genome after divergence. It was noted that two genes on scaffold 5 were mapped to the synteny block of *An. gambiae* chromosome 3R (*AsOR51*) and 3L (*AsOR54*). Similarly, the gene orthologs of *AsOR31*, *AsOR32*, *AsOR33*, *AsOR35* and *AsOR38* on scaffold 25 were localized on *An. gambiae* chromosome 2L and 2R. This syntenic pattern suggested that intrachromosomal translocation events may have taken place in the genome during the evolution of these two mosquitoes.

Ortholog identification and clustering of *An. sinensis* ORs

Ortholog clustering can be used to identify important patterns in gene conservation across diverse organisms and reveal unique gene sets that are important to one species. To this end, ortholog identification of genes among four mosquito genomes was performed using OrthoMCL against Diptera ortholog datasets. Thirty-four of 52 *AsORs* were categorized into 14 ortholog groups (OG), 36 of 73 *AgORs* were grouped into 13 OGs, 57 of 112 *CqORs* were categorized into 8 OGs, and 32 of 75 *AaORs* were categorized into nine OGs. Five OGs (OR49B, OR85D, ORCO, OR56A and OR67D) were shared by the four mosquito species (Fig. 2a). Eleven OGs were shared by phylogenetically close *An. gambiae* and *An. sinensis*, while seven OGs were shared by *Cx. quinquefasciatus*

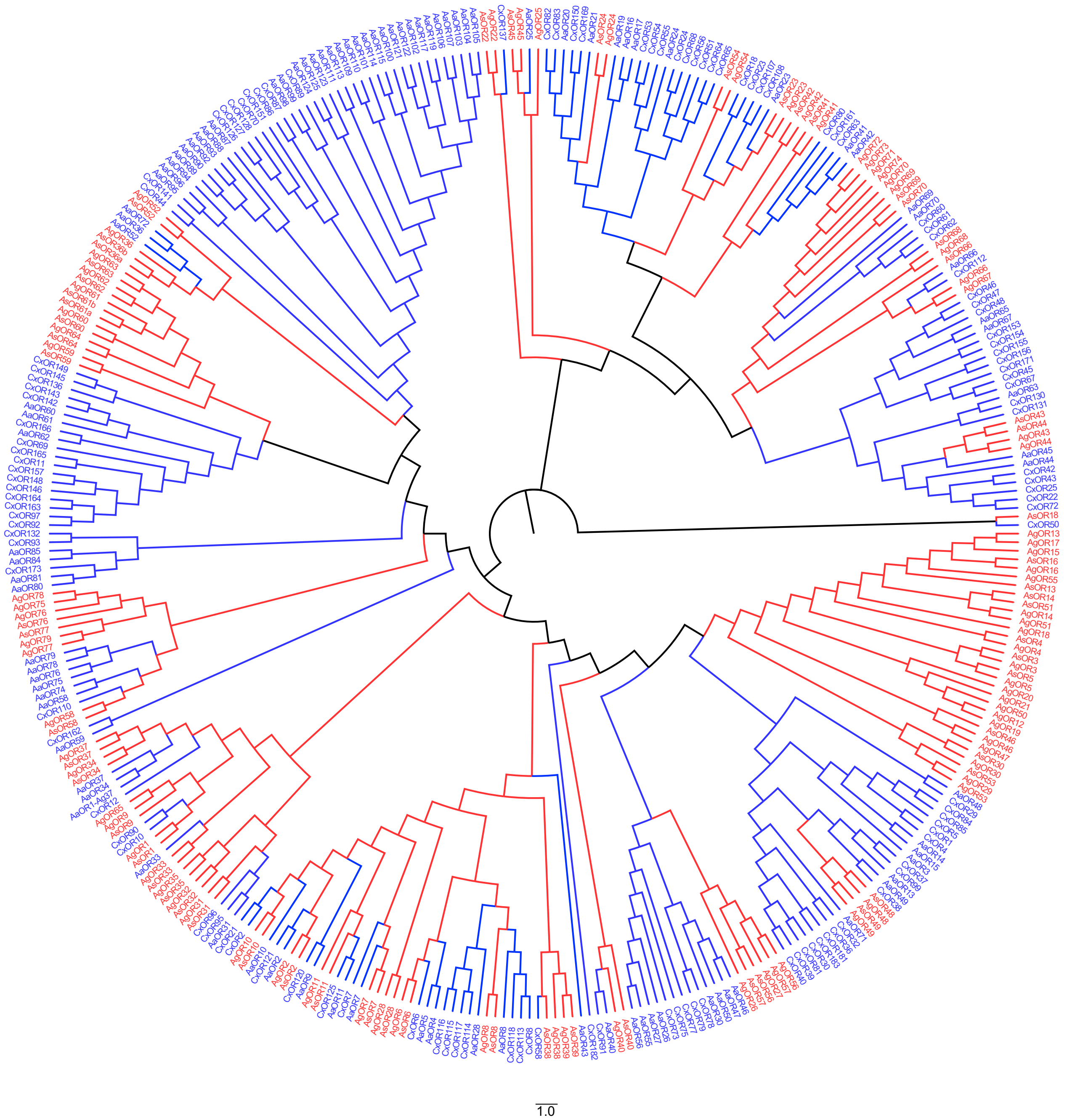

Fig. 3 Phylogenetic tree of OR proteins in *An. sinensis*, *An. gambiae*, *Ae. aegypti* and *Cx. quinquefasciatus*. Conserved domains of AsOR proteins were aligned using ClustalX, and the phylogenetic tree was constructed by RAxMLv8.2.0, using the maximum-likelihood method (1000 bootstrap) based on the best fit model of JTT+I+G

and *Aa. aegypti*. Interestingly, OR19A (*AsOR14*) and OR63A (*AsOR38*) were unique to *An. sinensis*. Previous studies showed that *Drosophila* favor egg-laying on citrus fruit, mediated by OR19A [57, 58]. In addition, some orthologous groups contained multiple paralogous genes in a species. For example, 13 AgORs, 11 AsORs, 39 CqORs and 17 AaORs were categorized into the OR67D group. It has been reported that communication between aggressive partners and their social environment in *D. melanogaster* is mediated by the 11-cis-vaccenyl acetate receptor, OR67D, in the trichoid sensilla [59–61]. The expansion of OR67D in four mosquito species may point

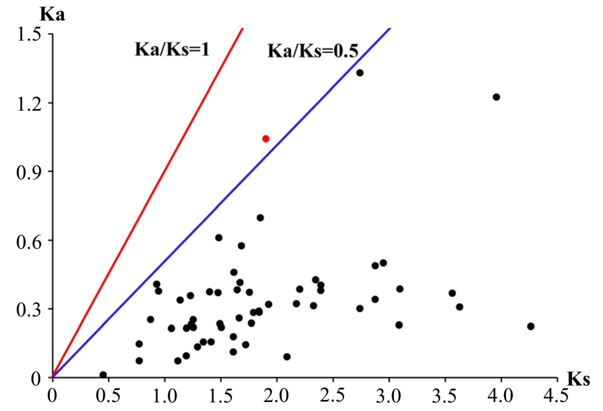

Fig. 4 Ka/Ks ratios of AsORs

to its importance in enhancing their pheromone perception and mate selection.

Phylogenetic and evolutionary analysis of *An. sinensis ORs*

The individual family members were extremely divergent and most exhibited from 7 to 28% amino acid identity. This is similar to the fly odorant receptors, which share 17 to 26% sequence identity at the amino acid level [55]. However, five gene sets had high identities: AsOR14/AsOR51 (88.9%), AsOR36a/AsOR36b (76.3%), AsOR61a/AsOR61b (85.7%), AsOR43/AsOR44 (84.1%) and AsOR56/AsOR57 (81.0%). Taken together, genomic clustering, conserved gene orientation and sequence similarly provided strong evidence that these genes may be expanded or duplicated from the same ancestor gene. Comparisons between different mosquito species revealed that the main feature of molecular evolution in the OR gene family was the expansion of subfamilies that are specific to the mosquito lineage. For example, there was a large subfamily of 22 *ORs* in *An. gambiae*, 85 *ORs* in *Cx. quinquefasciatus* and 104 *ORs* in *Ae. Aegypti*, with no close *An. sinensis* relatives (Additional file 2: Table S2). The phylogenetic tree showed that all AsORs had orthologous genes in *An. gambiae* or other mosquitoes (Fig. 3).

Gene expansion was also reflected in the high levels of variation in number of OR genes between different mosquitoes (Fig. 2b). The OR number in *An. sinensis* (59 genes, which included one pseudogene and one incomplete annotated gene) was less than that in *An. gambiae* (79 OR genes) [9]. However, the number of identified gene families, such as *CSP*, *OBP* and ionotropic glutamate receptor genes (iGluRs), was similar between the two species [38, 39, 41]. Thus, the *An. gambiae* OR gene family underwent gene expansion after divergence of the two species. Further research is needed into gene expansion. When compared with Culicinae mosquito species (158 ORs in *Ae. albopictus*, 131 ORs in *Ae. aegypti* and 112 ORs in *Cx. quinquefasciatus*) [9, 11, 13], the number of OR genes was reduced in Anophelinae mosquitoes. In addition to OR genes, *Ae. aegypti* and *Cx. quinquefasciatus* have significantly more chemosensory gene members of *CSP*, *OBP* and *iGluRs* than *An. sinensis* and *An. gambiae* [38, 41, 63, 64]. Both *An. sinensis* and *An. gambiae* are nighttime, indoor feeders, while *Ae. aegypti* and *Cx. quinquefasciatus* are daytime, aggressive outdoor feeders [39]. The expansion and contraction of the OR gene family may be linked to different host preferences or lifestyle habits [16, 19]. Therefore, there is a great need for systematic comparative and functional genomics studies on the OR repertoires of *An. sinensis* and other mosquitoes to further understand the olfactory-driven behaviors.

Estimation of the positive selection of *OR* genes in *An. sinensis*

The Ka/Ks ratio has been a popular parameter for genomic analysis of gene families and can provide insights into selective evolutionary pressures that act on genes. Of the 59 *AsORs*, 57 were orthologs, with counterparts in *An. gambiae*, but 23 genes in *An. gambiae* were absent from the *An. sinensis* genome. To better understand whether OR genes in *An. sinensis* and *An. gambiae* were subjected to different evolutionary constraints, the pairwise *Ka/Ks* was calculated for each ortholog group (Fig. 4). All *Ka/Ks* ratios were < 1, which implied that negative selection (purification selection) drove OR gene family evolution as the primary force in *An. sinensis* and *An. gambiae*. However, the *Ka/Ks* ratios of *AsOR18* and *AgOR38* were much higher than others, which indicated that they had undergone positive selective pressure. Olfactory genes of *An. sinensis*, such as *IRs* [41] and *OBPs* (unpublished data), were also subjected to purifying selection. A similar evolutionary pattern was observed in the *D. melanogaster* genome, in which purifying selection was the main selection pressure driving the diversities of *ORs*, *GRs* and *OBPs* [62].

Tissue-specific expression analysis by RNA-seq

Mapping and transcript prediction

Female mosquitoes use a combination of cues to find their vertebrate hosts and blood-feed [65]. Our long-term goal is to use genome-engineering techniques coupled with behavioral analysis to investigate the genetic and chemical ecological bases of host-seeking behaviors in *An. sinensis*. Exploration of the olfactory genes that sense these cues and their signaling pathways will help to explain the olfactory mechanisms by which mosquitoes track their hosts. *Anopheles sinensis* starts to blood-feed approximately 3 days after emergence. To fully understand the expression profiling of chemosensory genes in

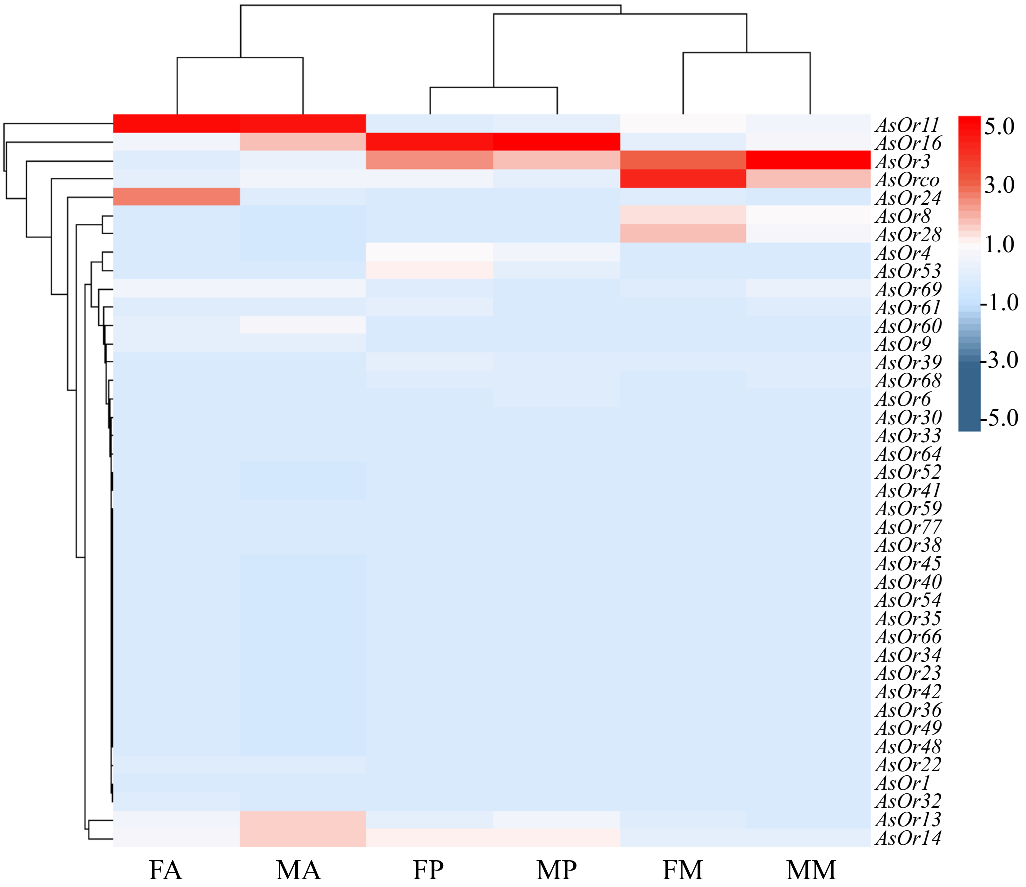

Fig. 5 Expression profiles of the OR genes in different tissues, based on RNA-seq. Expression levels of the OR genes in the six transcriptomes represented as heat plots, based on log-transformed FPKM values. Zero expression is represented by the lightest blue color

the olfactory appendages of *An. sinensis* at this developmental stage, triplicate transcriptomes were obtained from antenna, proboscis and the maxillary palp of adults of both sexes. General assembly statistics are summarized in Additional file 7: Table S4. The Illumina reads have been submitted to the Sequence Read Archive (SRA) at the NCBI (BioProject accession: PRJNA791160). The sequencing yielded a high number of clean reads, ranging from 42,950,993 to 57,016,567. The proportion of reads that successfully mapped to the *An. sinensis* genome assembly was high in all six transcriptomes, ranging from 88.96 to 91.77%. The combined Trinity assembly of all above transcriptomes resulted in 19,319 nonredundant putative transcripts, of which 1663 were novel. Principal component analysis (PCA) showed that the samples between the groups were scattered and the samples within the groups were clustered together (Additional file 8: Fig. S4). The cluster analysis also confirmed the overall quality of replicates in our RNA-seq procedure (Additional file 9: Fig. S5) and facilitated the identification of OR genes that represented transcriptome profile signatures of the different tissues and sexes.

Overall expression profiles of the chemosensory genes

Chemosensory genes have been identified across the genome of *An. sinensis* in the past few years. These genes have included 8 *CSPs* [38], 64 *OBPs* [39], 56 *iGluRs* [40–42], 59 *ORs* (in this study) and 77 *Grs* (unpublished data). Other chemosensory genes, such as *ODEs* and *SNMPs*, have not been reported. Based on the above results, a total of 146 chemosensory genes, which included 4 *CSPs*, 22 *OBPs*, 39 *ORs*, 39 *Irs* and 42 *GRs*, were identified in at least one tissue of 3-day-old adults of both sexes (Additional file 10: Table S5; Additional file 11: Table S6).

Gene expression profiles were analyzed by RNA-seq and quantified by FPKM values. The FPKM values of 49.32% of chemosensory genes ranged from 0 to 0.1, which meant that nearly half of the genes were not expressed or were expressed at low levels. Using FPKM > 0.1 as the threshold, 104 chemosensory genes

were expressed in at least one tissue and exhibited obvious tissue- and sex-specific or preferential expression patterns (Additional file 12: Table S7). We observed that most chemosensory genes were enriched or specifically expressed in the antennae. Similar results were also found in *Ae. albopictus* [25], *An. gambiae* [23], *Ae. aegypti* [66] and other insects [18, 67, 68]. The differences in expression profiles of chemosensory genes strongly suggested that the odor coding of antennae is far more complex and stronger than that of the maxillary palp or proboscis. Moreover, *OBPs*, *ORs* and *IRs* were mainly expressed in antennae and the maxillary palp, while *Grs* were mainly expressed in the proboscis (Additional file 11: Table S6). This is consistent with the well-established knowledge that the antennae and maxillary palps are the main olfactory organs, while the proboscis mainly processes gustatory information during food intake, oviposition and host recognition [69, 70]. In situ hybridization and single-sensillum electrophysiological recordings of fruit flies, mosquitoes and other insects indicated that neurons that express ORs and IRs respond to multiple volatile odors, which include many odors from humans [30, 71, 72], while *GRs* respond to a variety of stimuli, such as taste agents, pheromones, warmth and carbon dioxide [31, 73, 74]. These results confirmed that the mosquito antennae, maxillary palp and proboscis had large differences in chemical perception at the molecular, cellular and electrophysiological levels, although these appendages likely evolved from a common origin.

Expression profiles of OR genes

Of the 59 *ORs*, 39 were identified in the transcriptome data. Of these, 34 *ORs* were detectable in the antennae, 24 in the proboscis and 24 in the maxillary palp (Additional file 11: Table S6). Using FPKM > 0.1 as the threshold, nearly 72% of *OR* genes (28 *ORs*) were expressed in at least one of the analyzed tissues. Of these, 25 *OR* genes were expressed in antennae, 8 in the maxillary palp and 9 in the proboscis. Unidentified or unexpressed *ORs* may be tissue or development stage specific. For example, *AgOR37*, *AgOR40*, *AgOR52* and *AgOR58* of *An. gambiae* were specifically expressed in the larval stage [75]. Their orthologous genes (*AsOR37*, *AsOR40*, *AsOR52* and *AsOR58*) in *An. sinensis* were not expressed in 3-day-old adult mosquitoes, which suggested that they were likely to be larval-specific genes. The RT-PCR results confirmed that *AsOR37*, *AsOR40*, *AsOR52* and *AsOR58* were expressed in larvae but not in adults (Additional file 13: Fig. S6). Transcriptome analysis of the female mosquito legs, 3 days after emergence, showed that *AgOR31*, *AgOR34*, *AgOR41*, *AgOR43*, *AgOR44*, *AgOR48* and *AgOR54* were expressed in the legs (unpublished data) but not in the olfactory tissues. The *AgOR2* gene was highly enhanced in the female antennae of *An. gambiae* [23], which is narrowly tuned to a small set of aromatics, such as indole [30]. However, *AsOR2* was not detectable in adult *An. sinensis*. The *AsOR10* gene, which responds strongly to 3-methylindole, is directly involved in the identification of oviposition sites [43]. This was

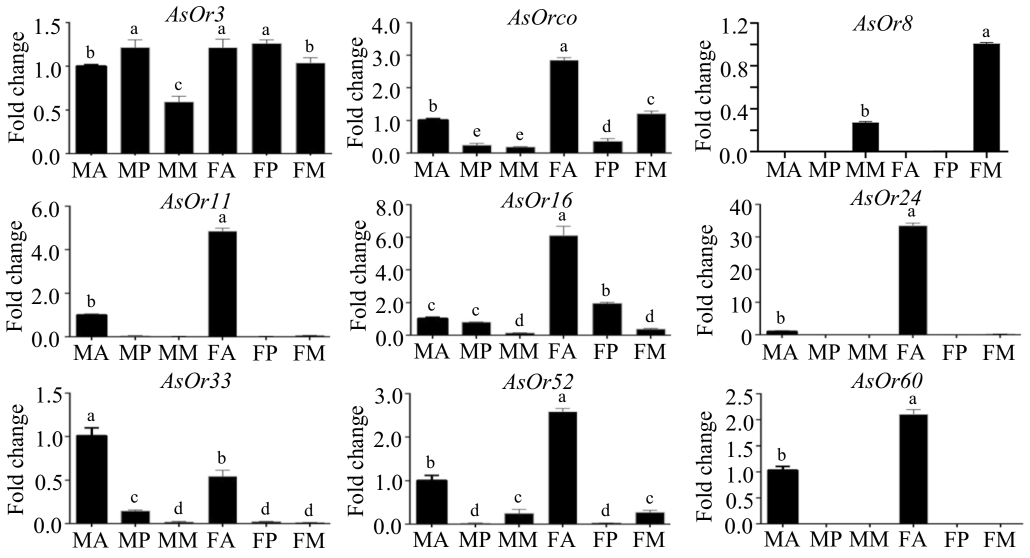

Fig. 6 Expression profiling of selected preferentially expressed AsOR genes in different tissues, based on RT-qPCR. The relative expression levels of nine genes in different tissues of adult mosquitoes of both sexes. Three biological and three technical replications were tested using RT-qPCRs. The expression ratios of each gene, normalized against the *Rps7* reference gene, are represented in bars, and the standard deviation is shown on the top of the bar, with different letters indicating significant differences determined by a one-way ANOVA ($P < 0.05$)

surprising as *AsOR10* was not detected in the adult olfactory tissues of *An. sinensis*.

Gene expression patterns of all 39 ORs in olfactory tissues were presented in a heatmap (Fig. 5). Apart from *AsOR3* and *AsOrco*, which were broadly expressed in all chemosensory tissues of both sexes, other genes showed obvious tissue-specific expression patterns. The majority of *AsORs* had higher FPKM values in the antennae than in other tissues, which was consistent with the gene expression pattern of their orthologous genes in *An. gambiae* [23]. Moreover, the relative levels of *AsOR* transcripts were much higher in the female antennae than in the male antennae. Previous studies revealed that *AsOrco* is required for establishing the function of OR complexes [43, 76], while the functions of *AsOR3* remain unknown. In the entire set of *AsOR* genes expressed in the antennae, *AsOR1*, *AsOR6*, *AsOR9*, *AsOR22*, *AsOR30*, *AsOR32*, *AsOR33*, *AsOR38*, *AsOR45*, *AsOR59*, *AsOR60*, *AsOR61*, *AsOR66*, *AsOR68*, *AsOR69* and *AsOR77* were antenna-specific expression genes. In particular, *AsOR6*, *AsOR45*, *AsOR66* and *AsOR68* were specifically expressed in female antennae, which suggested that they may have a role in female mosquito-specific behaviors, such as host-seeking and oviposition.

Compared with the antennae, fewer OR genes were expressed in the maxillary palp and proboscis, and they were expressed at a lower level (Additional file 7: Table S4). The *AsOR8* and *AsOR28* genes were specifically expressed in the maxillary palp, which was consistent with the expression pattern of their orthologous genes (*AgOR8* and *AgOR28*) in *An. gambiae* [23, 31] and *AaOR8* of *Ae. aegypti* [11]. Previous studies have shown that olfactory receptor neurons in the maxillary palps of *Ae. aegypti* [73], *Cx. quinquefasciatus* [77] and *An. gambiae* [31] modulated the response to octenol and CO_2. For *An. gambiae* and *Ae. aegypti*, OR8 showed a strong response to 1-octen-3-ol, which is a human volatile that is strongly attractive to mosquitoes [30–32]. Therefore, whether AsOR8 could also respond to 1-octen-3-ol and CO_2 needs to be studied further. Among the nine genes expressed in the proboscis, *AsOR4* was specifically expressed in female mosquitoes, but its function is unclear.

To validate the gene expression patterns observed in the above RNA-seq experiments, nine *AsOR* genes were selected, and RT-qPCRs were performed. Generally, there was a high correlation between gene expression levels given by both approaches. The expression patterns of nine *AsOR* genes were significantly different between tissues (one-way ANOVA: *AsOR3*: F (5, 12) = 174.6, $P < 0.0001$; *AsOrco*: F (5, 12) = 4144, $P < 0.0001$; *AsOR8*: F (5, 12) = 14,595, $P < 0.0001$; *AsOR11*: F (5, 12) = 4082, $P < 0.0001$; *AsOR16*: F (5, 12) = 2111, $P < 0.0001$; *AsOR24*: F (5, 12) = 1791, $P < 0.0001$; *AsOR33*: F (5, 12) = 1141, $P < 0.0001$; *AsOR52*: F (5, 12) = 439.8, $P < 0.0001$; *AsOR60*: F (5, 12) = 4895, $P < 0.0001$), which was consistent with the results obtained by RNA-seq (Fig. 6). These results also confirmed the reliability of our gene expression data.

Conclusions

In this study, we identified and characterized 59 putative OR members in *An. sinensis*. We also examined the expression profiles of these genes in various chemosensory tissues. This was the first comprehensive study of ORs in *An. sinensis*, which is a major malaria vector in China and countries in Southeast Asia. Compared to the OR family of *An. gambiae*, the number of OR genes identified in *An. sinensis* was significantly lower. Whether this difference is caused by the contraction or expansion of ORs genes after divergence of the two species remains to be studied further. Analysis of RNA-seq data showed that *AsORs* exhibited obvious tissue- and sex-specific expression patterns. The great majority of *AsORs* were strongly expressed in the antennae. Moreover, the relative levels of *AsORs* were significantly higher in female antennae than in male antennae, which indicated that odor sensitivity is likely to be enhanced in females. We combined results from previous studies to speculate on the functions of some OR genes. However, this still requires validation by further behavioral, molecular and electrophysiological studies. The results of this study provided genetic and behavioral research directions and targets for future vector control.

Abbreviations

ORs: Odorant receptors; GRs: Gustatory receptors; IRs: Ionotropic receptors; OBPs: Odorant-binding proteins; CSPs: Chemosensory proteins; SNMPs: Sensory neuron membrane proteins; ODEs: Odorant degrading enzymes; NPC2: Niemann–Pick protein C2; Orco: Odorant receptor co-receptor; HMM: Hidden Markov model; MW: Molecular weight; pI: Isoelectric point; FPKM: Fragments per kilobase of transcript per million mapped reads; Aa: Amino acids; GPCRs: G-protein-coupled receptors; OG: Ortholog groups; SRA: Sequence read archive.

Supplementary Information

The online version contains supplementary material available at https://doi.org/10.1186/s13071-022-05259-x.

Additional file 1: Table S1. Primers used in this study.

Additional file 2: Table S2. Homology relationships of *Anopheles sinensis* with three mosquitoes and *Drosophila melanogaster* ORs.

Additional file 3: Table S3. The amino acid and nucleic acid sequences of *Anopheles sinensis* OR genes.

Additional file 4: Figure S1. The motifs of AsORs obtained from the MEME analysis.

Additional file 5: Figure S2. Sequence logo plot of transmembrane region of AsORs.

Additional file 6: Figure S3. Phylogenetic and gene structure analysis of AsOR genes. The unrooted maximum-likelihood phylogenetic tree was constructed on conserved domain under the model of JTT + I + G with 1000 bootstrap replicates. Gene structures were analyzed using the online server Gene Structure Display Server. Boxes in green were exons. Solid lines represent introns. Number 0, 1 and 2 represent intron phases.

Additional file 7: Table S4. Statistics of RNA-seq based sequencing, assembling and functional annotation for *Anopheles sinensis*.

Additional file 8: Figure S4. S4 RNA-seq correlation check. FA: female antennae; FP: female proboscis; FM: female maxillary palps; MA: male antennae; MP: male proboscis; MM: male maxillary palps.

Additional file 9: Figure S5. The correlation of 6 transcriptomes was analyzed using Euclidean Distance. FA: female antennae; FP: female proboscis; FM: female maxillary palps; MA: male antennae; MP: male proboscis; MM: male maxillary palps.

Additional file 10: Table S5. The differential expression of chemosensory genes in the six transcriptomes.

Additional file 11: Table S6. The chemosensory genes identified from the six transcriptomes.

Additional file 12: Table S7. Statistical table of chemosensory genes in different expression levels.

Additional file 13: Figure S6. The semi quantitative PCR results of OR genes. The total RNA was extracted from larvae or 3-day- old adults and was reverse transcribed into the first-strand cDNA using the SuperScript III RT Kit (Invitrogen, Carlsbad, CA, USA). These cDNAs were used as templates and semi quantitative PCR was performed. The primers were designed using Primer 5.0 and are listed in Additional file 1: Table S1. M: marker; L: larvae; A: adult.

Acknowledgements
The authors would like to thank Yongjie Zhang and Chengyin He for mosquito rearing.

Author contributions
ZB and BC conceived and designed the study. ZB, ZR, XF, YJ, QL, SH and JJ performed data analysis and drafted the manuscript. All authors read and approved the final manuscript.

Funding
This work was supported by the National Natural Science Foundation of China (31572332), the Program of Natural Science Foundation of Chongqing (cstc2019jcyj-msxmX0242) and the Venture & Innovation Support Program for Chongqing Overseas Returnees. The funders had no role in study design, data collection and analysis, decision to publish, or preparation of the manuscript.

Availability of data and materials
The Illumina reads of the six olfactory tissues have been deposited in Sequence Read Archive at the NCBI under BioProject accession PRJNA791160. All data generated or analyzed in this study were included in this MS and the additional files. Any related data can be available from the corresponding author on reasonable request.

Declarations

Ethics approval and consent to participate
Not applicable.

Consent for publication
Not applicable.

Competing interests
The authors declare that they have no competing interests.

Received: 12 January 2022 Accepted: 29 March 2022
Published online: 23 April 2022

References

1. Sinka ME, Bangs MJ, Manguin S, Chareonviriyaphap T, Patil AP, Temperley WH, et al. The dominant *Anopheles* vectors of human malaria in the Asia-Pacific region: occurrence data, distribution maps and bionomic précis. Parasit Vectors. 2011;4:89.
2. Feng X, Zhang S, Huang F, Zhang L, Feng J, Xia Z, et al. Biology, bionomics and molecular biology of *Anopheles sinensis* Wiedemann 1828 (Diptera: Culicidae), main malaria vector in China. Front Microbiol. 2017;8:1473.
3. Pan JY, Zhou SS, Zheng X, Huang F, Wang DQ, Shen YZ, et al. Vector capacity of *Anopheles sinensis* in malaria outbreak areas of central China. Parasit Vectors. 2012;5:136.
4. Zhang S, Guo S, Feng X, Afelt A, Frutos R, Zhou S, et al. Anopheles vectors in mainland China while approaching malaria elimination. Trends Parasitol. 2017;33:889–900.
5. Zwiebel LJ, Takken W. Olfactory regulation of mosquito–host interactions. Insect Biochem Mol Biol. 2004;34:645–52.
6. Rützler M, Zwiebel LJ. Molecular biology of insect olfaction: recent progress and conceptual models. J Comp Physiol A Neuroethol Sens Neural Behav Physiol. 2005;191:777–90.
7. Zhu J, Guo MB, Ban LP, Song LM, Liu Y, Pelosi P, et al. Niemann–Pick C2 proteins: a new function for an old family. Front Physiol. 2018;9:52.
8. Benton R, Sachse S, Michnick SW, Vosshall LB. Atypical membrane topology and heteromeric function of *Drosophila* odorant receptors in vivo. PLoS Biol. 2006;4: e20.
9. Hill CA, Fox AN, Pitts RJ, Kent LB, Tan PL, Chrystal MA, et al. G protein-coupled receptors in *Anopheles gambiae*. Science. 2002;298:176–8.
10. Robertson HM, Wanner KW. The chemoreceptor superfamily in the honey bee, *Apis mellifera*: expansion of the odorant, but not gustatory, receptor family. Genome Res. 2006;16:1395–403.
11. Bohbot J, Pitts RJ, Kwon HW, Rützler M, Robertson HM, Zwiebel LJ. Molecular characterization of the *Aedes aegypti* odorant receptor gene family. Insect Mol Biol. 2007;16:525–37.
12. Wanner K, Anderson A, Trowell S, Theilmann D, Robertson HM, Newcomb R. Female-biased expression of odourant receptor genes in the adult antennae of the silkworm, *Bombyx mori*. Insect Mol Biol. 2007;16:107–19.
13. Arensburger P, Megy K, Waterhouse RM, Abrudan J, Amedeo P, Antelo B, et al. Sequencing of *Culex quinquefasciatus* establishes a platform for mosquito comparative genomics. Science. 2010;330:86–8.
14. Zhan S, Merlin C, Boore JL, Reppert SM. The monarch butterfly genome yields insights into long-distance migration. Cell. 2011;147:1171–85.
15. You M, Yue Z, He W, Yang X, Yang G, Xie M, et al. A heterozygous moth genome provides insights into herbivory and detoxification. Nat Genet. 2013;45:220–5.
16. Engsontia P, Sangket U, Chotigeat W, Satasook C. Molecular evolution of the odorant and gustatory receptor genes in lepidopteran insects: implications for their adaptation and speciation. J Mol Evol. 2014;79:21–39.
17. Chen XG, Jiang X, Gu J, Xu M, Wu Y, Deng Y, et al. Genome sequence of the Asian tiger mosquito, *Aedes albopictus*, reveals insights into its biology, genetics, and evolution. Proc Natl Acad Sci USA. 2015;112:E5907–15.
18. Wang Z, Yang P, Chen D, Jiang F, Li Y, Wang X, et al. Identification and functional analysis of olfactory receptor family reveal unusual characteristics of the olfactory system in the migratory locust. Cell Mol Life Sci. 2015;72:4429–43.
19. Zhou X, Rokas A, Berger SL, Liebig J, Ray A, Zwiebel LJ. Chemoreceptor evolution in Hymenoptera and its implications for the evolution of eusociality. Genome Biol Evol. 2015;7:2407–16.
20. Karpe SD, Jain R, Brockmann A, Sowdhamini R. Identification of complete repertoire of *Apis florea* odorant receptors reveals complex orthologous relationships with *Apis mellifera*. Genome Biol Evol. 2016;8:2879–95.
21. He P, Wang MM, Wang H, Ma YF, Yang S, Li SB, et al. Genome-wide identification of chemosensory receptor genes in the small brown planthopper, *Laodelphax striatellus*. Genomics. 2020;112:2034–40.

22. Marinotti O, Cerqueira GC, de Almeida LG, Ferro MI, Loreto EL, Zaha A, et al. The genome of *Anopheles darlingi*, the main neotropical malaria vector. Nucleic Acids Res. 2013;41:7387–400.
23. Pitts R, Rinker D, Jones P. Transcriptome profiling of chemosensory appendages in the malaria vector *Anopheles gambiae* reveals tissue-and sex-specific signatures of odor coding. BMC Genomics. 2011;12:271.
24. Leal WS, Choo YM, Xu P, da Silva CS, Ueira-Vieira C. Differential expression of olfactory genes in the southern house mosquito and insights into unique odorant receptor gene isoforms. Proc Natl Acad Sci USA. 2013;110:18704–9.
25. Lombardo F, Salvemini M, Fiorillo C, Nolan T, Zwiebel LJ, Ribeiro JM, et al. Deciphering the olfactory repertoire of the tiger mosquito *Aedes albopictus*. BMC Genomics. 2017;18:770.
26. Kaupp UB. Olfactory signaling in vertebrates and insects: differences and commonalities. Nat Rev Neurosci. 2010;11:188–200.
27. DeGennaro M, McBride CS, Seeholzer L, Nakagawa T, Dennis EJ, Goldman C, et al. Orco mutant mosquitoes lose strong preference for humans and are not repelled by volatile DEET. Nature. 2013;498:7455.
28. McBride CS, Baier F, Omondi AB, Spitzer SA, Lutomiah L, Sang R, et al. Evolution of mosquito preference for humans linked to an odorant receptor. Nature. 2014;515:222–7.
29. Liu H, Liu T, Xie L, Wang X, Deng Y, Chen CH, et al. Functional analysis of Orco and odorant receptors in odor recognition in *Aedes albopictus*. Parasit Vectors. 2016;9:363.
30. Carey AF, Wang G, Su CY, Zwiebel LJ, Carlson JR. Odorant reception in the malaria mosquito *Anopheles gambiae*. Nature. 2010;464:66–71.
31. Lu T, Qiu YT, Wang G, Kwon JY, Rutzler M, Kwon HW, et al. Odor coding in the maxillary palp of the malaria vector mosquito *Anopheles gambiae*. Curr Biol. 2007;17:1–12.
32. Bohbot JD, Dickens JC. Characterization of an enantioselective odorant receptor in the yellow fever mosquito *Aedes aegypti*. PLoS ONE. 2009;4: e7032.
33. Pellegrino M, Steinbach N, Stensmyr MC, Hansson BS, Vosshall LB. A natural polymorphism alters odour and DEET sensitivity in an insect odorant receptor. Nature. 2011;478:511–4.
34. Xu P, Choo YM, De La Rosa A, Leal WS. Mosquito odorant receptor for DEET and methyl jasmonate. Proc Natl Acad Sci USA. 2014;111:16592–7.
35. Xu P, Zeng F, Bedoukian RH, Leal WS. DEET and other repellents are inhibitors of mosquito odorant receptors for oviposition attractants. Insect Biochem Mol Biol. 2019;113: 103224.
36. Grant GG, Estrera RR, Pathak N, Hall CD, Tsikolia M, Linthicum KJ, et al. Interactions of DEET and novel repellents with mosquito odorant receptors. J Med Entomol. 2020;57:1032–40.
37. Zhou D, Zhang D, Ding G, Shi L, Hou Q, Ye Y, et al. Genome sequence of *Anopheles sinensis* provides insight into genetics basis of mosquito competence for malaria parasites. BMC Genomics. 2014;15:42.
38. Mei T, He ZB, Wang XT, Wang TT, Chen B. Genome-wide identification and characterization of genes of the chemosensory protein (CSP) family in *Anopheles sinensis* (Diptera: Culicidae). Acta Entomol Sinica. 2017;60:136–47 (**In Chinese**).
39. He X, He ZB, Zhang YJ, Zhou Y, Xian PJ, Qiao L, et al. Genome-wide identification and characterization of odorant-binding protein (OBP) genes in the malaria vector *Anopheles sinensis* (Diptera: Culicidae). Insect Sci. 2016;23:366–76.
40. Wang TT, Hao YJ, He ZB, Mei T, Chen B. Characteristics and classification position of the ionotropic receptor genes IR8a and IR25a in four vector mosquito species of medical importance. Acta Entomol Sinica. 2017;60:379–99 (**In Chinese**).
41. Wang TT, Si FL, He ZB, Chen B. Genome-wide identification, characterization and classification of ionotropic glutamate receptor genes (iGluRs) in the malaria vector *Anopheles sinensis* (Diptera: Culicidae). Parasit Vectors. 2018;11:34.
42. Li J, Chen Q, Man Y, Pei D, Wu W. Variant Ionotropic Receptors are Expressed in the Antennae of *Anopheles sinensis* (Diptera: Culicidae). Biochem Genet. 2019;57:571–82.
43. Liu H, Liu L, Cheng P, Huang X, Gong M. An odorant receptor from *Anopheles sinensis* in China is sensitive to oviposition attractants. Malar J. 2018;17:348.
44. Yang Z, Nielsen R. Estimating synonymous and nonsynonymous substitution rates under realistic evolutionary models. Mol Biol Evol. 2000;17:32–43.
45. Yang Z. PAML 4: phylogenetic analysis by maximum likelihood. Mol Biol Evol. 2007;24:1586–91.
46. Katoh K, Kuma K, Toh H, Miyata T. MAFFT version 5: improvement in accuracy of multiple sequence alignment. Nucleic Acids Res. 2005;33:511–8.
47. Stamatakis A. RAxML version 8: a tool for phylogenetic analysis and post-analysis of large phylogenies. Bioinformatics. 2014;30:1312–3.
48. Kim D, Langmead B, Salzberg SL. HISAT: a fast spliced aligner with low memory requirements. Nat Methods. 2015;12:357–60.
49. Trapnell C, Williams BA, Pertea G, Mortazavi A, Kwan G, van Baren MJ, et al. Transcript assembly and quantification by RNA-Seq reveals unannotated transcripts and isoform switching during cell differentiation. Nat Biotechnol. 2010;28:511–5.
50. Pertea M, Pertea GM, Antonescu CM, Chang TC, Mendell JT, Salzberg SL. StringTie enables improved reconstruction of a transcriptome from RNA-seq reads. Nat Biotechnol. 2015;33:290–5.
51. Wang L, Feng Z, Wang X, Wang X, Zhang X. DEGseq: an R package for identifying differentially expressed genes from RNA-seq data. Bioinformatics. 2010;26:136–8.
52. Benjamini Y, Yekutieli D. The control of the false discovery rate in multiple testing under dependency. Ann Stat. 2001;29:1165–88.
53. Livak KJ, Schmittgen TD. Analysis of relative gene expression data using real-time quantitative PCR and the 2−ΔΔCT method. Methods. 2001;25:402–8.
54. Robertson HM, Warr CG, Carlson JR. Molecular evolution of the insect chemoreceptor gene superfamily in *Drosophila melanogaster*. Proc Natl Acad Sci USA. 2003;100:14537–42.
55. Vosshall LB, Amrein H, Morozov PS, Rzhetsky A, Axel R. A spatial map of olfactory receptor expression in the *Drosophila* antenna. Cell. 1999;96:725–36.
56. Kroeze WK, Sheffler DJ, Roth BL. G-protein-coupled receptors at a glance. J Cell Sci. 2003;116:4867–9.
57. Dweck HK, Ebrahim SA, Kromann S, Bown D, Hillbur Y, Sachse S, et al. Olfactory preference for egg laying on citrus substrates in *Drosophila*. Curr Biol. 2013;23:2472–80.
58. Ebrahim SA, Dweck HK, Stökl J, Hofferberth JE, Trona F, Weniger K, et al. *Drosophila* avoids parasitoids by sensing their semiochemicals via a dedicated olfactory circuit. PLoS biol. 2015;13: e1002318.
59. Ha TS, Smith DP. A pheromone receptor mediates 11-cis-vaccenyl acetate-induced responses in *Drosophila*. J Neurosci. 2006;26:8727–33.
60. Wang L, Anderson DJ. Identification of an aggression-promoting pheromone and its receptor neurons in *Drosophila*. Nature. 2010;463:227–31.
61. Schneider J, Atallah J, Levine JD. Social structure and indirect genetic effects: genetics of social behaviour. Biol Rev. 2017;92:1027–38.
62. Tunstall NE, Sirey T, Newcomb RD, Warr CG. Selective pressures on *Drosophila* chemosensory receptor genes. J Mol Evo. 2007;64:628–36.
63. Croset V, Rytz R, Cummins SF, Budd A, Brawand D, Kaessmann H, et al. Ancient protostome origin of chemosensory ionotropic glutamate receptors and the evolution of insect taste and olfaction. PLoS Genet. 2010;6: e1001064.
64. Manoharan M, Chong MNF, Vaïtinadapoulé A, Frumence E, Sowdhamini R, Offmann B. Comparative genomics of odorant binding proteins in *Anopheles gambiae*, *Aedes aegypti*, and *Culex quinquefasciatus*. Genome Biol Evol. 2013;5:163–80.
65. Raji JI, DeGennaro M. Genetic analysis of mosquito detection of humans. Curr Opin Insect Sci. 2017;20:34–8.
66. Matthews BJ, McBride CS, DeGennaro M, Despo O, Vosshall LB. The neurotranscriptome of the *Aedes aegypti* mosquito. BMC Genomics. 2016;17:32.
67. Andersson MN, Videvall E, Walden KK, Harris MO, Robertson HM, Löfstedt C. Sex- and tissue-specific profiles of chemosensory gene expression in a herbivorous gall-inducing fly (Diptera: Cecidomyiidae). BMC Genomics. 2014;15:501.
68. Olafson PU, Saski CA. Chemosensory-related gene family members of the horn fly, *Haematobia irritans irritans* (Diptera: Muscidae), identified by transcriptome analysis. Insects. 2020;11:816.
69. Maekawa E, Aonuma H, Nelson B, Yoshimura A, Tokunaga F, Fukumoto S, et al. The role of proboscis of the malaria vector mosquito *Anopheles stephensi* in host-seeking behavior. Parasit Vectors. 2011;4:10.
70. Choo YM, Buss GK, Tan K, Leal WS. Multitasking roles of mosquito labrum in oviposition and blood feeding. Front Physiol. 2015;6:306.
71. Hallem EA, Ho MG, Carlson JR. The molecular basis of odor coding in the *Drosophila* antenna. Cell. 2004;117:965–79.

72. Benton R, Vannice KS, Gomez-Diaz C, Vosshall LB. Variant ionotropic glutamate receptors as chemosensory receptors in *Drosophila*. Cell. 2009;136:149–62.
73. Grant AJ, Wigton BE, Aghajanian JG, O'Connell RJ. Electrophysiological responses of receptor neurons in mosquito maxillary palp sensilla to carbon dioxide. J Comp Physiol. 1995;177:389–96.
74. Jones WD, Cayirlioglu P, Grunwald Kadow I, Vosshall LB. Two chemosensory receptors together mediate carbon dioxide detection in *Drosophila*. Nature. 2006;445:86–90.
75. Xia Y, Wang G, Buscariollo D, Pitts RJ, Wenger H, Zwiebel LJ. The molecular and cellular basis of olfactory-driven behavior in *Anopheles gambiae* larvae. Proc Natl Acad Sci USA. 2008;105:6433–8.
76. Sato K, Pellegrino M, Nakagawa T, Nakagawa T, Vosshall LB, Touhara K. Insect olfactory receptors are heteromeric ligand-gated ion channels. Nature. 2008;452:1002–6.
77. Syed Z, Leal WS. Maxillary palps are broad spectrum odorant detectors in *Culex quinquefasciatus*. Chem Senses. 2007;32:727–38.
78. Hao YJ, Zou YL, Ding YR, Xu WY, Yan ZT, Li XD, et al. Complete mitochondrial genomes of *Anopheles stephensi* and *An. dirus* and comparative evolutionary mitochondriomics of 50 mosquitoes. Sci Rep. 2017;7:7666.

Publisher's Note

Springer Nature remains neutral with regard to jurisdictional claims in published maps and institutional affiliations.

Ready to submit your research? Choose BMC and benefit from:

- fast, convenient online submission
- thorough peer review by experienced researchers in your field
- rapid publication on acceptance
- support for research data, including large and complex data types
- gold Open Access which fosters wider collaboration and increased citations
- maximum visibility for your research: over 100M website views per year

At BMC, research is always in progress.

Learn more biomedcentral.com/submissions

BMC
