# Supplementary material for: Effects of antennal segments defects on blood-sucking behavior in Aedes albopictus
Source: PLoS One. 2023 Aug 10;18(8):e0276036. doi: 10.1371/journal.pone.0276036 (PMC10414602; doi:10.1371/journal.pone.0276036)
Supplement: Attachment — Submitted filename: response to reviewers.docx [file pone.0276036.s003.docx]

**Response to Reviewers**

We thank the editor and reviewers for their time, feedback, and constructive suggestions. We have addressed the reviewers’ comments point by point below. Changes have been documented in the manuscript in highlight text. We have also added a few citations to the manuscript.

**Reviewer #1: It is an interested study and provided some interested findings for further understanding the antenna with sensors /receptors function for continuing blood feeding. The results are not surprised because the antenna with sensors is for a long range purpose to find the odor and hosts. There are many studies on the host-seeking and blood feeding behaviors of Aedes albopictus in China and other Asian countries in the 1980's and 1990's. The host-seeking and blood feeding is a complicate issue, not only the receptors on the antenna, also, other parts of organs, such as papa, proborese , and tarsal, mating status, distance between hosts and mosquitoes, and other many factors influence on the host-seeking and blood- feeding behaviors. The following questions and comments may assist the authors for further revision:**

**1. Provide the strain of Aedes albopictus (Hunan?) and more information about the laboratory colony (how long of the colony you and they have and what kind of regular blood resource used even if this species is an opportunity feeder).**

A strain of Ae. albopictus used in this study was isolated from the wild in Changsha, Hengyang, Chenzhou, and Yueyang Counties, Hunan Province, which was obtained from the Center for Disease Control of Hunan Province (China). The colony in our laboratory has been in culture since 2016. Adult female mosquitoes were fed on the blood of anesthetized mouse in regularly. We have added this to the section of “Mosquito Rearing for Stock Propagation”

**2. Have you sealed the antenna after you cut? What was the speed for orientation, probing time, and** **how long for the blood engorgement, compared with non-cut individual mosquitoes?**

We did not think about sealing the antenna, and also did not know how to seal the wound. We just reserved the healing time of 2 days based on the observations in our pre-trial. No records were made for orientation speed, probing time, and blood engorgement time, but it was observed that the orientation speed and probing time depended on the number of antenna segments cut compared to uncut individual mosquitoes. In our pre-trials, the localization and detection abilities of mosquitoes with the missing distal antennal segments were almost the same as those of untreated mosquitoes. However, with the increase of missing antennae, the flight activity, orientation speed, and probing time of mosquitoes decreased gradually.

The reviewer's question really triggered our thinking, because the antennae become thinner from the base to the end, and the more antennae segments cut, the larger the wound was. Is the decline of mosquito blood-sucking behavior also related to the increase of wound? Whether wound sealing is needed to promote healing is something to consider. Thank the reviewer for the questions. We have also added these thoughts to the discussion section.

**3. Provide more details about human volunteers due to the variation by individual.**

Since the variable in this test is the antennae length of mosquitoes, we need to select volunteers as long as they meet the screening criteria and can follow the requirements strictly. A female teacher (33 years old, married and childless) with 165cm height and 51kg weight was finally selected. She claimed that she did not smoke or drink, and the attraction to mosquitoes is moderate. Because of the interesting of this study and could tolerate mosquito bites. She decided to volunteer, which she eventually did it.

**4. Italic for all** **scientific name of the species in text and references.**

We have italicized all scientific name of the species in text and references.

**5. More discussion about the receptors/sensors. There were many studies about the olfactory sensors, also, single sense cell function/role detected by EAG ..**

Thanks to the reviewer for reminding us that the paper lacks sufficient discussion. We have discussed related issues in the revised version.

**6. There were several publications about** **the receptors of antenna of Aedes albopictus in China. You need to search for the Chinese literatures and understand where you stand and start. Huang, XJ, et al. 1991. Studies on the ultra-structures of antennal receptors of Aedes albopictus. Chinese Journal of Vector Biology and Control Vol. 2 (suppl.): 29-32. Xue RD. 1991. Studies on the olfactory behavior of Periplaneta americana, Musca domestica, and Aedes albopictus (NSF project summary). Chin J Vector Biol Control. Vol. 2 (suppl):5-9. Also, there is a reviewing article about this subject. Xue RD. 2016. Host-seeking and blood feeding behavior of Aedes albopictus. Technical Bulletin of the Florida Mosquito Control Association, Vol. 10:2-13. You may find this article from the ResearchGate or the Florida Mosquito Control Association website at** [**www.yourfmca.org**](http://www.yourfmca.org)

Thank the reviewers for providing us with a lot of learning materials, including reminding us to pay attention to Chinese literature. We learned a lot from them and discussed much in the section of “Discussion and Conclusions”.

**Reviewer #2: Nice research, good interpretation of data. However, two points: One, this is not frontline research. I have attached a file the stand of the research of today. With the molecular tools we have today, we should use them to get a clear idea where we could interact genetically. The field is so hot these days that it is hard to keep up. However, that is the name of the game. Two, by cutting the antennae short one does cut all sensory system short. To attribute a change of behavior to olfaction may not be correct. One would have to show that mechanoreception has not been altered.**

**I can see that a more thorough literature review would improve the manuscript. For example, similar research has been conducted with other small animals (e.g., zooplankton, fruitflies) with similar results. This would then give a better platform for their observed data. --> major edit**

Thanks to the editor's suggestion, the idea of this paper comes from the observation in the laboratory that mosquitoes with incomplete antennae can still successfully suck blood. We guessed that there might be some protective mechanism in the 13 antennal flagellomeres of mosquitoes. We want to know how much antennal flagellomeres loss will affect its sucking behavior, and we want to show you the result of this idea. We've actually done something at the molecular level based on these results, but we've run into some difficulties and haven't made any progress yet. Anyhow, we have rethought a lot in this process and added a lot of content to the "Discussion and Conclusion" section.
