# Supplementary material for: Effects of antennal segments defects on blood-sucking behavior in Aedes albopictus
Source: PLoS One. 2023 Aug 10;18(8):e0276036. doi: 10.1371/journal.pone.0276036 (PMC10414602; doi:10.1371/journal.pone.0276036)
Supplement: Attachment — Submitted filename: response to reviewers 2.docx [file pone.0276036.s004.docx]

**Response to Reviewers**

We thank the editor and reviewers for their time, feedback, and constructive suggestions. We have addressed the reviewers’ comments point by point below. Changes have been documented in the manuscript in highlight text. We have also added a few citations to the manuscript.

**Reviewer #2: So, here my evaluation of the revised manuscript.**

**All little points are fine, the English is somewhat charming but understandable.**

**The value of this manuscript is so/so. Very old-fashioned research when compared with the research done on Drosophila, for example.**

**Here an abstract of similar research:**

**Functional Interaction Between Drosophila Olfactory Sensory Neurons and Their Support Cells**

**by Sinisa Prelic et al --- https://doi.org/10.3389/fncel.2021.789086**

**Insects detect volatile chemicals using antennae, which house a vast variety of olfactory sensory neurons (OSNs) that innervate hair-like structures called sensilla where odor detection takes place. In addition to OSNs, the antenna also hosts various support cell types. These include the triad of trichogen, tormogen, and thecogen support cells that lie adjacent to their respective OSNs. The arrangement of OSN supporting cells occurs stereotypically for all sensilla and is widely conserved in evolution. While insect chemosensory neurons have received considerable attention, little is known about the functional significance of the cells that support them. For instance, it remains unknown whether support cells play an active role in odor detection, or only passively contribute to homeostasis, e.g., by maintaining sensillum lymph composition. To investigate the functional interaction between OSNs and support cells, we used optical and electrophysiological approaches in Drosophila. First, we characterized the distribution of various supporting cells using genetic markers. By means of an ex vivo antennal preparation and genetically-encoded Ca2+ and K+ indicators, we then studied the activation of these auxiliary cells during odor presentation in adult flies. We observed acute responses and distinct differences in Ca2+ and K+ fluxes between support cell types. Finally, we observed alterations in OSN responses upon thecogen cell ablation in mature adults. Upon inducible ablation of thecogen cells, we notice a gain in mechanical responsiveness to mechanical stimulations during single-sensillum recording, but a lack of change to the neuronal resting activity. Taken together, these results demonstrate that support cells play a more active and responsive role during odor processing than previously thought. Our observations thus reveal that support cells functionally interact with OSNs and may be important for the extraordinary ability of insect olfactory systems to dynamically and sensitively discriminate between odors in the turbulent sensory landscape of insect flight.**

**Reading this abstract we can see that the discussion about "wound healing" is way out considering what the researchers above published.**

**In this context, the results of the authors are fine as results. One has to start somewhere. However, they have to connect with literature like above to make it clear, that their research is the first step to more targeted research where one would use their step as step one and continue with research as above as the guiding research for further meaningful research.**

**I realize that this field is very competitive and to get a clear step forward is hard to achieve.**

**So, based on this, it is your decision.**

**One, the research is not up to the scientific level of your journal;**

**or**

**Two, for a review one would need another round of editing with a clear appreciation of the research done on other species (see literature cited in above).**

**I would prefer solution TWO. These researchers would gain a lot of new knowledge and would move in the direction of modern olfactory research. However, you may not have the pages for it (--> send it to an appropriate journal).**

We would like to thank the reviewers for giving us the opportunity to revise our manuscript. We would also like to thank the reviewers for providing us with references. We have read this and other related literatures carefully and learned a lot from them. 1. Learned the potential role of antennal auxiliary cells in the sense of smell in mosquitoes and other insects, which increased our attention to these types of cell in the future; 2. Learned the methods of studying the function of ORNs in antennae ex vivo, which are more advanced, objective and intuitive compared with our methods. The direct imaging of cations in living cells can indeed more intuitively observe the response of different parts of antennae to odors. For the above two knowledge points and some other olfactory research methods, we added descriptions in the discussion section.

In this study, we used a behavioral approach to explore the role of antennal segments in mosquitoes' blood-sucking behavior. Since the behavior itself is affected by many factors, we just wanted to try to eliminate the interference of pain factors, so we took the healing time of the wound into consideration. Although we did not know whether the healing of the wound would affect the mosquito's behavior, we did so, and we talked about it. In contrast, cationic imaging of antennae ex vivo observes physiological responses of cells that may not be affected by pain. But anyway, as the reviewer said, our simple and extensive study is the first step of the follow-up research, Thanks to this process, we have learned a lot. Modern methods of olfactory research are also the direction of our efforts.

**Reviewer #3: The investigation has been done on very obvious and general objective: whoever study basic moth part and feeing mechanisms can expect antenatal segment efects will affect blood sucking behaviour. However, the specific study on mosquito antennal flagellomeres and particularly is an enhancement of nowledge. Which flagellomere may affect their blood-feeding behavior, and through rationally designed behavioral experiments, we found that the 6th and 7th flagellomeres on Aedes albopictus antenna are important in the olfactory detection of host searching. Meanwhile, the morphology and distribution of sensilla on each antenna flagellomere were also analysed and discussed in this paper. Over all It is an interesting study and provided some new findings for further understanding the antenna with sensors /receptors function. However, the results are nothing rather, along the expected line.**

**My specific comments are as follow:**

**Abstract: Too general statement has been provided in the introductory sentences in abstract The blood-feeding transmits extremely harmful infections including malaria, yellow fever, dengue fever, and other arboviruses, making mosquitoes one of the most harmful creatures to human health.**

Thanks to the review's suggestion, we have revised the abstract and marked the changes.

**Methods:
Authors have neither performed proper** **sealing of the antenna, nor studied orientation activity of the antenna, this implication some line of future studies should be explained.**

have neither performed proper sealing of the antenna, nor studied orientation activity of the antenna, which is attributed to our limited knowledge reserve. Thank you for the reviewer's prompt. We also learned and thought during the revision process, and discussed in the section of shortcomings and limitations.

**Repetition of the scientific name Aedes albopictus
Write ones’ full name then abbreviate form as Ae albopictus**

Except the first appearance of the scientific name (Aedes albopictus), we have changed the following full name into an abbreviation form as Ae albopictus.

**Discussion: There should be some lines of discussion on the host-seeking and blood feeding behaviour as this is a complicated issue, not only the receptors on the antenna, also, other parts of organs, such as proborese , and tarsal, mating status, distance between hosts and mosquitoes, etc. are also important determinant of the behaviour so proper discussion explaining implication of these results on vector transmission, blood sucking an knowledge enhancement behavioural attributes of host seeking blood feeing should be explained.**

According to the suggestions of reviewers, in the discussion section, we have focused on the mosquito sensory system and its role in host location, the factors affecting blood-sucking behavior and the methods of mosquito olfaction research.
