# Supplementary material for: Effects of antennal segments defects on blood-sucking behavior in Aedes albopictus
Source: PLoS One. 2023 Aug 10;18(8):e0276036. doi: 10.1371/journal.pone.0276036 (PMC10414602; doi:10.1371/journal.pone.0276036)
Supplement: Attachment — Submitted filename: response to reviewers 3.docx [file pone.0276036.s005.docx]

**Response to Reviewers**

We thank the editor and reviewers for their time, feedback, and constructive suggestions. We have addressed the reviewers’ comments point by point below. Changes have been documented in the manuscript in highlight text.

**Reviewer #2: Yes, do catch up with today's olfaction research. Your starting point is fine. However, for progress we need more detailed information that we can compare with results from other insects.**

Thanks for the reviewer's encouraging comment! Under the inspiration of the reviewer, tracking and comparing the results of other insects will be our long-term research plan, as we are conducting molecular research based on this study, real-time information will help us with our future research.

**Reviewer #3: Revision has been done. quality of the Ms is better and understandable now. However the manuscript need minor revision before acceptance. Following clarification amendment are required**

Thanks for the reviewer's encouraging comment!

**1. The revised version has an additional author hope the** **procedure of authorship modification has been followed.**

Thanks for the reviewer's reminder, the added author made important contribution in our revision process, we will add her in accordance with the authorship modification procedures required by the journal.

**2. The introductory sentences in abstract The blood-feeding transmits extremely harmful infections including malaria, yellow fever, dengue fever, and other arboviruses, making mosquitoes one of the most harmful creatures to human health. Is too general statement and may be deleted from abstract?**

We have deleted the statement from the abstract.

**3. Introduction: line 21-24: After mating, female mosquitoes.…. blood feeding. Shorten the** **sentence.**

We have revised the sentence with a shorter statement in Line 25-26: Female mosquitoes require blood meals to complete their oogenesis, during which they release pathogens into the hosts or become infected themselves.

**4. line 41: This paper attempts…molecular level; Could be rewritten as** **This paper combines morphological and behavioural studies to determine the functional regions of olfactory organ of mosquitoes to narrow down the range of research targets at cellular and molecular levels.**

We thank the reviewer for the suggestion. We have revised the manuscript accordingly in Line 44-46.

**5. Material and methods: Line 136-140; The physical characteristics of** **volunteer could be depicted as pointers like;** **Age: 33, Sex: female, Height: 165cm.. and so on.**

**Was there only one participant out of the 23 sign ups?**

The reviewer’s point is well taken. We have depicted the physical characteristics as pointers in Line 138-142.

Yes, among the 23 registrants, there is indeed only one participant. Since this is a preliminary study on the effect of antennal segments on the blood-sucking behavior of female mosquitoes, we hope that the research variable is only the antennal segments of female mosquitoes, so we tried to keep other conditions unchanged, including blood donors. Setting up only one participant and selecting the most suitable participant from multiple registrants was our initial design. However, if more different volunteers had joined the experiment to increase the selectivity of mosquitoes, would different results be obtained? This is also what we are thinking about currently, and it may also be where we need to improve in the future. As for the number of participants, we have mentioned in the " Shortcomings and Limitations" part of the manuscript.

**6. Line 158-159: Grammatical error;** **Participants were aware of the content and purpose of the study but were unaware of the test order.**

We are sorry for our grammar errors. We have made corrections in Line 160-161.
